# The Shift to Bio-Based Auxiliaries in Textile Wet Processing: Recent Advances and Industrial Potential

**DOI:** 10.3390/molecules30194016

**Published:** 2025-10-08

**Authors:** Maria L. Catarino, Filipa Sampaio, Luísa Pacheco, Ana L. Gonçalves

**Affiliations:** 1 CITEVE, Centro Tecnológico das Indústrias Têxtil e do Vestuário de Portugal, Rua Fernando Mesquita, 2785, 4760-034 Vila Nova de Famalicão, Portugal; mcatarino@citeve.pt (M.L.C.); fsampaio@citeve.pt (F.S.); lpacheco@citeve.pt (L.P.); 2 2C2T, Centre for Textile Science and Technology, University of Minho, 4800-058 Guimarães, Portugal; 3 LEPABE, Laboratory for Process Engineering, Environment, Biotechnology and Energy, Faculty of Engineering, University of Porto, Rua Dr. Roberto Frias, 4200-465 Porto, Portugal; 4 ALiCE, Associate Laboratory in Chemical Engineering, Faculty of Engineering, University of Porto, Rua Dr. Roberto Frias, 4200-465 Porto, Portugal

**Keywords:** bio-based compounds, biopolymers, enzymes, microbial agents, plant- and agrowaste-based agents, sustainability, textile wet processes

## Abstract

The textile industry is among the most resource-intensive sectors, heavily dependent on water, energy, and synthetic chemicals, particularly in wet processing stages such as desizing, scouring, bleaching, dyeing, printing, and finishing. Conventional practices generate vast amounts of contaminated wastewater, posing severe risks to ecosystems and human health. In recent years, growing environmental concerns and stricter regulations have accelerated the search for sustainable alternatives. Biotechnology offers promising solutions, including enzymes, biopolymers, plant- and agrowaste-derived materials, and microbial metabolites, which can replace conventional auxiliaries and reduce the ecological footprint of textile processing. This review provides a structured overview of recent advances in bio-based compounds applied across different stages of textile wet processing. Applications are critically assessed in terms of performance, efficiency, environmental benefits, and potential for industrial adoption. Current limitations, future outlooks, and examples of commercially available products are also discussed. By highlighting the most recent progress, this review underscores the potential of bio-based innovations to support the transition toward more sustainable and resource-efficient textile manufacturing.

## 1. Introduction

The global textile industry is one of the most resource-intensive sectors, playing a vital role in economies worldwide while simultaneously placing considerable pressure on the environment. As consumer demand for textiles continues to rise, so does the urgency to address the environmental impacts of textile manufacturing. Among the most pressing challenges are the excessive consumption of water, energy, and hazardous chemicals, particularly in textile wet processing [1].

Traditionally, textile wet processing, which comprises essential stages such as desizing, scouring, bleaching, dyeing, printing and finishing, has relied heavily on water-intensive methods. This dependency has significant environmental consequences, primarily due to the discharge of large volumes of contaminated wastewater. For example, a study reported that a textile facility producing 1,438 tonnes of cotton fabric annually generated approximately 91 litres of wastewater per kilogram of fabric, amounting to 131 million litres of wastewater produced in a single year [2]. A broad range of chemical products, including sodium hydroxide (NaOH), hydrogen peroxide (H_2_O_2_), acids, bases, salts, synthetic dyes, detergents and softeners, are conventionally employed in these processes [3]. While these chemicals are essential for achieving fabric quality and performance, their extensive use results in heavily contaminated effluents that pose significant risks to freshwater ecosystems, soil quality, and ultimately, human health.

In response to growing environmental concerns and increasing regulatory frameworks, the textile industry is actively exploring more sustainable alternatives. Among promising solutions, the integration of biotechnology by leveraging enzymes, biopolymers, plant- and agrowaste-based materials, and microbial metabolites, offers eco-friendlier substitutes for conventional chemical treatments. These bio-based innovations not only can reduce the environmental impact of textile processing but also contribute to improved process efficiency and lower water and energy consumption.

This review highlights recent advancements in replacing conventional synthetic auxiliaries with bio-based alternatives in textile wet processing. To provide a clear and structured overview, the article is organised into different categories of bio-based compounds—namely enzymes, biopolymers, and other bioactive agents. For each of these categories, their application potential across different stages of textile wet processing is discussed and evaluated. Within this framework, the review focuses specifically on developments from the last five years, assessing their potential for industrial adoption, performance, current limitations, and future outlook. In addition to recent research studies, whenever possible, companies already commercialising such bio-based products are also identified. While the emphasis is placed on new developments, readers seeking detailed background information on conventional methods, chemical auxiliaries, and associated wastewater composition are encouraged to examine our previously published review article [3], which provides a comprehensive breakdown of these primary aspects. The previous review also provides a detailed overview of resource efficiency strategies, such as bath ratio optimisation and energy-saving approaches, which complement the bio-based innovations emphasised in this review.

## 2. Enzymes

**Figure 1:**
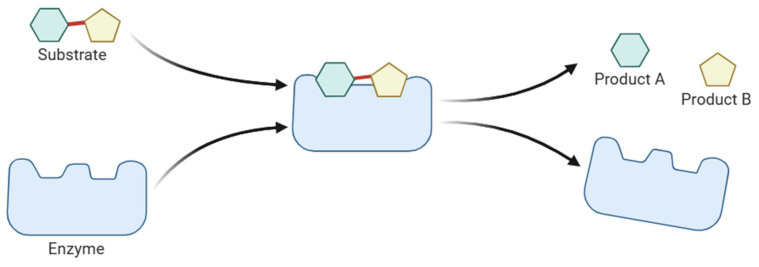
Mechanism of enzyme-substrate interaction.

Enzymes are specialised proteins that act as biocatalysts, accelerating chemical reactions by lowering the activation energy required. They are primarily derived from biological sources, such as plants, animal tissues and microbes [4,5], which makes them inherently biodegradable and non-toxic. A key feature of enzymes is their high specificity, enabling them to interact with a particular substrate to convert it into a new molecule. This degree of selectivity minimises side reactions and reduces the production of unwanted by-products [6]. Enzymatic reactions typically occur under mild conditions, such as lower temperatures and neutral pH, which can result in significant energy savings and reduced environmental impact [5]. These characteristics have led to widespread adoption of enzymes in various industrial sectors. Figure 1 provides a schematic representation of enzymatic catalysis, illustrating the specific interaction between enzyme and substrate.

In the textile industry, enzymes offer a promising approach to align with increasing environmental regulations and sustainability targets. Due to their compatibility with textile fibres, enzymes are particularly useful during wet processing. In pre-treatment processes, for example, enzymatic treatments can enable effective impurity removal while preserving the structural integrity of the fabrics and supporting the implementation of environmentally responsible practices [7]. Thus, enzymatic treatments represent a compelling compromise between industrial performance and environmental sustainability.

Enzymes are broadly classified as hydrolases, oxidoreductases, isomerases, transferases, lyases, and ligases [8,9]. In the context of textile wet processing, hydrolases and oxidoreductases are predominantly employed. Hydrolases catalyse the hydrolysis of various chemical bonds, while oxidoreductases facilitate oxidation-reduction reactions [8]. A wide range of enzymes is currently used in textile applications, including amylases, lipases, pectinases, cellulases, proteases, cutinases, xylanases, catalases, laccases, and peroxidases. Each of these enzymes play a specific role in distinct stages of textile wet processing. Table 1 provides their classification, associated processing steps and a summary of their role in textile industry applications.

### 2.1. Desizing

Yarns are typically sized with starch and wax-like products prior to weaving to reduce friction and minimise breakage probability. However, conventional size removal requires the use of harsh chemicals such as NaOH at around 95 °C. These methods are not only unsustainable but can also damage the fabric structure [3].

In contrast, amylases have been successfully applied for decades to hydrolyse starch-based sizes, without affecting textile fibres, thereby preserving fabric integrity [12]. Amylases are a great example of the significance of enzyme implementation on an industrial scale [11,12]. Amylase desizing is considered effective, low-cost and a sustainable method for the textile processing industry, contributing to 30% of the industrial enzyme market [9,13].

The typical enzymatic desizing process involves three main steps: (i) application of hot water to swell and gelatinise the starch film, promoting enzyme penetration; (ii) hydrolysis of the starch with amylase; and (iii) a subsequent washing step to remove the solubilised residues [14]. Standard process conditions for enzymatic desizing are in the range of 5.5–6.5 pH and 30–60 °C [5,8], which are considerably gentler compared to conventional chemical treatments.

There are three main types of amylases used in industrial processes: α-amylase, which randomly cleaves internal [1,4] bonds in starch; β-amylase, which cleaves [1,4] bonds from the non-reducing ends; and γ-amylase, which hydrolyses both [1,4] and [1,6] bonds [6]. Among these, α-amylase is the most used for textile desizing due to its rapid and random cleavage of internal bonds, which facilitates efficient solubilisation and removal of starch-based sizing products [9].

### 2.2. Scouring

Scouring is the essential process of removing non-cellulosic impurities naturally present in textile fibres, such as pectins, proteins, fats and waxes, which make the fibres hydrophobic and thus unsuitable for effective dyeing and finishing [15]. For successful downstream processing, fibres must exhibit hydrophilic properties, making scouring essential to ensure adequate wettability and absorbency. Traditionally, scouring is performed using boiling temperatures, NaOH and surfactants to break down these impurities. While effective, this is a harsh method that often leads to fibre damage, intensive water consumption for rinsing, and the production of contaminated wastewater [8]. In contrast, enzyme-assisted scouring offers a more sustainable alternative. It operates under milder conditions, preserves fibre integrity, and significantly reduces harmful chemical use. Studies show that enzymatic treatments can lower biological oxygen demand (BOD), chemical oxygen demand (COD) and total dissolved solids (TDS) in generated effluents values by over 50% compared to conventional scouring [5].

As detailed in Table 1, several enzymes can be used during enzyme-assisted scouring. They remove specific impurities, allowing for a tailored and environmentally friendlier approach. Pectinases degrade pectin, the primary non-cellulosic component in cotton fibres, facilitating the release of waxes and other bound impurities [12,15]. Proteases hydrolyse protein-based contaminants into amino acids [5]. Lipases break down surface oils and fats [15]. Cutinases target cutin, a waxy polymer on the fibre surface [12]. Xylanases hydrolyse xylan, a major hemicellulose component, aiding in the removal of residual non-cellulosic matter, especially for bast fibres [12]. By selectively targeting these impurities, enzyme-assisted scouring induces structural changes that expose more polar hydroxyl groups on the fibre surface, thereby enhancing hydrophilicity [15].

### 2.3. Bleaching

In conventional bleaching, H_2_O_2_ is the most widely used bleaching agent. However, this process typically requires temperatures between 90 and 120 °C and long processing times, resulting in elevated energy consumption and increased fibre damage [13]. Unlike desizing and scouring steps, where enzymes can effectively replace chemical treatments with comparable results, enzyme-assisted bleaching has not yet achieved performance levels competitive with conventional chemical methods.

Nonetheless, certain enzymes show promise in supporting or enabling low-impact bleaching approaches. For instance, laccases can assist in colour removal by oxidising flavonoid phenolic compounds [12], while peroxidases contribute to low intensity bleaching by catalysing the chemical reduction of H_2_O_2_ [5]. An emerging enzymatic strategy involves indirect bleaching systems employing glucose oxidase. This enzyme generates H_2_O_2_ in situ by oxidising glucose, eliminating the need to add chemical H_2_O_2_ and offering a more controlled and milder bleaching process that reduces fibre degradation [13]. After conventional H_2_O_2_ bleaching, 10–15% of H_2_O_2_ often remains in the fabric, where it can degrade cellulose and compromise fibre strength [8]. To mitigate this, catalase is increasingly used to decompose residual H_2_O_2_ into water and oxygen, reducing the need for intensive rinsing, saving water and energy, and lowering H_2_O_2_ levels in resulting effluents [12].

### 2.4. Dyeing

Conventional dyeing typically relies on synthetic dyes and auxiliary products that are harmful to the environment and tend to be discharged in dyeing effluents, typically known for their high colour intensity, elevated conductivity from salt-based electrolytes, the presence of toxic auxiliaries, and low biodegradability [3]. In contrast, enzyme-assisted dyeing, particularly using laccases, offers a promising sustainable alternative to reduce the use of synthetic dyes and auxiliary products in dyeing, while improving de biodegradability of resulting effluents.

Laccases are oxidoreductase enzymes that catalyse the oxidation of aromatic dye precursors by removing electrons. This process generates free radicals and oxidised aromatic compounds, many of which absorb visible light, thereby developing colour [13]. These reactive dye intermediates can then bind directly to textile fibres, enabling in situ dye formation and eliminating the need for pre-manufactured dyes and chemicals [16]. Laccase-assisted dyeing typically occurs under room or mildly elevated temperatures (between 50 and 60 °C), significantly reducing energy consumption and lowering overall environmental impact of the dyeing process [17,18].

This method is particularly well-suited for protein fibres, such as wool and silk, due to their high contents in amino, thiol, carboxyl and phenolic groups, which readily react with oxidised dye intermediates [17]. These interactions form strong covalent bonds, contributing to good dye fixation and durability. In the study performed by Polak et al. [19], wool was dyed using laccase and a synthetic oligomer precursor formed from 2-amino-3-methoxybenzenesulfonic acid and 2-aminonaphthalenesulfonic acid. The dyeing process was carried out at 100 °C for 30 min, resulting in good colour fastness and the additional benefit of antibacterial activity against *Staphylococcus aureus*. Another study used synthetic phenol, benzene and naphthol derivatives as precursors, enabling the production of a wide range of colours on wool. The resulting shades included brown and orange at pH 6, dark browns at pH 3, and greens at pH 8 [16]. In the same study, Nylon 6,6, a synthetic polyamide fibre rich in animo groups, responded effectively to the same laccase dyeing process, yielding pink and orange at pH 6, lilac at pH 3, and blue at pH 8. These examples highlight the versatility of laccase-assisted dyeing in creating diverse shades using only enzymes and precursors.

In contrast to protein-based fibres, cotton, composed primarily of cellulose, contains mainly hydroxyl groups, which offer fewer reactive sites for covalent bonding, resulting in weaker dye-fibre interactions [18]. Nevertheless, cotton dyed with natural precursors extracted from *Portulaca oleracea* L. stems and leaves at 90 °C for 3 h achieved very good to excellent washing and rubbing fastness, and good light fastness [20]. Similarly, extracts from St. John’s wort and white onion skins applied at 80 °C for 2 h resulted in good washing and rubbing fastness with fair light fastness, an enhancement over natural dyeing without laccase [21]. However, promising approaches to overcome dye-fibre interaction limitations under milder conditions have emerged. One approach involved a chitosan pre-treatment, which, when followed by laccase-assisted dyeing using synthetic hydroquinone as a precursor at 55 °C for 6 h, led to significantly improved dye uptake and excellent colour fastness properties on cotton fabrics [17]. Another promising method involves the use of ultrasounds, which enhanced colour strength (K/S) and dyeing efficiency on cotton when using a natural phenolic precursor at 60 °C for 3 h, outperforming conventional laccase dyeing in the same conditions [18].

Laccase-assisted dyeing offers a sustainable alternative to conventional synthetic dyeing processes by enabling in situ dye formation under environmentally friendlier conditions. Current studies comparing the cost of laccase-assisted dyeing with reactive dyeing show that water and energy consumption are significantly lower in the enzymatic process. However, the high cost of dyeing precursors remains a major barrier to widespread industrial adoption. Notably, when excluding dye precursor costs, laccase-assisted dyeing can reduce overall dyeing expenses by up to 55% compared to reactive dyeing, while also generating effluents that require only biological treatment [18]. Alternatives such as precursors of natural origin can benefit from the application of this method. Although a gap between research and industrial implementation exists, the advancements outlined here underscore the potential of enzyme-assisted dyeing as a viable pathway toward more sustainable textile manufacturing.

### 2.5. Finishing

Textile finishing comprises the final steps in fabric preparation with the objective of conferring desirable properties such as softness, dimensional stability, and surface smoothness, ensuring that the product meets both consumer expectations and industry standards. Without these treatments, fabrics may be prone to rough textures, shrinkage, excessive pilling, or felting, which can compromise both appearance and performance. Traditionally, mechanical and chemical finishing methods, such as singeing for pilling control or resin treatments for shrink resistance, are employed to address these issues [13]. However, these processes are often resource-intensive, involving high water and energy consumption as well as potentially harmful chemicals [3]. In this context, enzyme-assisted finishing emerges as a sustainable alternative, offering enhanced fabric quality while significantly reducing the environmental impact of textile processing.

For cotton, cellulases are commonly employed to reduce surface hairiness and remove pills, effectively smoothing the fabric’s surface [5]. They are widely used in industrial settings due to their stability and efficacy [9], with companies such as Creative Enzymes (Shirley, NY, USA) and Christeyns (Ghent, Belgium) offering specialised formulations. Other cellulosic fibres, such as linen, were compatible with cellulase treatment. In the study performed by Koksharov et al. [22], low-temperature immersion and impregnation methods demonstrated the potential to replace conventional chemical polishing for linen fabrics.

In contrast, wool benefits from protease-based antifelting treatments, which are typically applied before or during dyeing [5]. Wool’s characteristic felting shrinkage arises from the interlocking of fibre scales during washing. Proteases mitigate this by partially hydrolysing the outer cuticle proteins, thereby reducing fibre entanglement, minimising shrinkage, and improving surface smoothness [23]. However, common proteases exhibit limited activity on keratin, which is resistant to enzymatic hydrolysis due to its disulfide bonds [24]. To overcome this, several approaches are being developed, including the use of keratinolytic proteases, such as proteinase K [23], keratinolytic-cutinase systems [25], pre-treatments with biosurfactants [26] or thioglycolic acid compounds [27], and modified proteases combined with L-cysteine [28]. These innovations improve antifelting performance and dyeability while preserving wool’s mechanical integrity.

Another important application of enzyme-assisted finishing is the treatment of denim garments. Newly produced denim tends to be stiff and rigid, requiring additional processing to achieve the desired aesthetic finish. Conventionally, this involves mechanical abrasion and chemical bleaching, processes that are energy and water intensive, and release harmful effluents [29]. For example, non-enzymatic bleach-based treatments used to decolourise indigo can damage the fibres and contribute to environmental pollution. To address these concerns, enzyme-assisted finishing has been developed as an alternative and is already being commercialised by several textile auxiliary manufacturers. For instance, Rudolf Duraner (Bursa, Turkey) offers the Rucolase^®^ product range, specifically designed to achieve stone-wash effects on indigo-dyed denim using enzymatic treatments. Another example is Novonesis (Lyngby, Denmark), a company created through the combination of expertise and resources from Novozymes (Bagsværd, Denmark) and Chr. Hanse (Hørsholm, Denmark), with a dedicated focus on biosolutions. It offers enzyme-based products such as Novoprime^®^, Denimax^®^ and Cellusoft^®^ for denim abrasion. Cellulases can be used to remove excessive indigo dye by weakening fibre-dye interactions, softening the fabric in the process [12]. Laccases, in combination with chemical mediators, can break down indigo dye selectively by producing free radicals that oxidise the dye molecules without damaging the fabric [29]. These methods reduce water consumption and eliminate the need for harsh oxidants, offering a more environmentally friendly approach. However, it is worth noting that newer dry-finishing techniques, such as laser and ozonation treatments, are gaining attention for their considerably lower environmental impact [3]. Despite this, enzyme-based processes remain a viable, accessible, and greener option for facilities unable to implement cutting-edge technologies, bridging the gap toward more sustainable denim production.

### 2.6. Optimising Enzymatic Treatments

Despite the clear environmental and functional advantages of enzymatic processes in textile wet processing, several limitations have hindered their full industrial integration. Enzymes typically operate within narrow ranges of temperature and pH, making them vulnerable to inactivation under the harsh conditions commonly used in conventional textile processing [6,30]. Moreover, relatively high cost of commercial enzymes, coupled with their limited reusability, poses economic challenges for large-scale implementation [24].

To address these issues, several strategies have been explored. One promising approach is the use of thermophilic and extremophilic enzymes, derived from microorganisms that thrive under extreme conditions, capable of withstanding higher temperatures and broader pH ranges. This enables enzymatic treatments that are more compatible with existing industrial workflows, particularly in processes like desizing, scouring, and bleaching, which benefit from elevated temperatures. For instance, while typical enzymatic desizing occurs at 30–60 °C and 5.5–6.5 pH [5,8], extremophile-derived amylases demonstrated optimal activity at 80 °C and pH 9, aligning more closely with conventional manufacturing conditions [30]. Although milder conditions are more appealing from a sustainability standpoint, initial adoption by the manufacturers may depend on enzyme systems that function under already established parameters. Once enzymatic treatments are better integrated, a gradual transition to milder, more energy-efficient processes can be encouraged to achieve further environmental goals.

Advances in genetic engineering and microbial biotechnology have also enabled the production of more robust and cost-effective enzymes. Using inexpensive substrates and fermentation-enhancing techniques such as ultrasound, researchers have improved enzyme yields and stability [31]. An example is the bio-valorisation of desizing effluent, which is typically rich in starch. *Trichoderma reesei*-derived α-amylase produced using starch effluent, demonstrated optimal desizing activity at 80 °C, a temperature well-suited with industrial application conditions [32]. Similarly, fungal cellulase was also efficiently produced from cotton-polyester textile waste [33], while sugarcane bagasse, a widely available agro-industrial residue, was also used to extract cellulase enzymes with bio-finishing effects comparable with commercial alternatives [34]. In parallel, the development of enzyme immobilisation techniques and one-bath, multi-enzyme systems represents a major step forward. These innovations not only enhance treatment specificity and control but also allow for enzyme recovery and reuse—reducing waste and overall process cost.

The following subsections explore two key approaches to optimise enzymatic application in textile processing: multi-enzyme and one-bath systems, and enzyme immobilisation strategies.

#### 2.6.1. Combined Enzyme Systems and One-Bath Solutions

A promising approach in enzymatic textile processing is the integration of multiple enzymes with complementary functions into a single bath. A multi-enzyme strategy can significantly reduce water and energy consumption while also reducing processing time.

One common application is the combination of enzymes to optimise a particular wet processing step to achieve comparable or improved results to conventional techniques. For instance, during desizing, because starches can vary in structure and composition, a combination of enzymes may be more effective in achieving complete size removal [35]. Furthermore, a study conducted by Zhang et al. [14] demonstrated that non-starch components, such as proteins and lipids, can hinder enzymatic hydrolysis by physically wrapping up or combining with sizes. The inclusion of lipases was shown to have synergistic effects, by effectively hydrolysing triglycerides and waxy lubricants commonly found in size formulations, thereby facilitating the removal of hydrophobic components [6].

For scouring, a combined pectinase–cutinase bath applied at 37 °C on cotton accelerated cuticle removal and increased fibre wettability compared to pectinase alone [36]. Nevertheless, the use of low temperature resulted in incomplete removal of waxy residues, negatively affecting wettability. To address this, another study demonstrated that enzymatic scouring using pectinase at 55 °C for 30 min, followed by a 70 °C rinse with low surfactant concentration (0.2 g L^−1^), yielded wettability comparable to conventional scouring [15]. A xylanase–pectinase blend on sisal fibres at 50 °C enhanced brightness by over 23% and tensile strength by 11%, outperforming conventional scouring. For jute, a cellulase–pectinase–xylanase blend with ultrasound assistance at 55 °C for just 10 min significantly improved water absorption with increased tensile strength and comparable wettability [37,38].

In denim finishing, combining amylase, cellulase, and laccase not only improved aesthetics, by fading and softening the fabric, but also significantly reduced environmental impact. One study reported that wastewater from enzymatic denim processing required nearly four times less oxygen for pollutants degradation compared to conventional finishing wastewaters [39].

These examples highlight the potential of enzyme combinations to enhance specific textile treatments. Building upon this strategy, one-bath and multienzyme solutions have emerged as an innovative next step, enabling the combination of different processing steps into a single, integrated treatment.

In the study performed by El-Fiky et al. [40], a one bath solution was proposed for simultaneous scouring and dyeing of wool, using a lipase and an anionic dye at 70 °C and pH 4.5 for 2 h. The resulting dye exhaustion was comparable to conventional two-step processes, with the advantage of significantly reducing water and energy consumption. Although total processing time was similar, conventional scouring requires a thorough rinsing step that adds hidden time and water demand—both avoided in the one-bath method. Similarly, another study applied a one-bath desizing and dyeing process on viscose, achieving improved K/S and comparable colour fastness. The process used a maximum of 80 °C over 175 min for overall treatment, in contrast to the conventional method that required 100 °C and 6 h [41]. A more complex example involved a three-enzyme system with glucoamylase, pectinase and glucose oxidase applied on cotton at 50 °C for 60 min, followed by natural dyeing with an increase in temperature to 80 °C for 90 min. Glucoamylase breaks down starch from sizing into glucose, which is then converted into H_2_O_2_ via glucose oxidase. This in situ generation of H_2_O_2_ replaces the need for added chemical bleaching agents. Following enzymatic treatment, dyeing with plant-based dyes was conducted. The inclusion of ultrasound further enhanced desizing efficiency, fabric whiteness, K/S, and colour fastness properties [42]. This system demonstrates not only the feasibility of integrating desizing, scouring, and pre-bleaching into a single step, but also the potential to replace conventional bleaching chemicals with enzymatically produced agents, dramatically reducing chemical input, energy consumption, and wastewater treatment needs. Moreover, the breakdown of hydrogen peroxide after its function is complete contributes to lower water consumption and reduced post-treatment steps.

Together, these findings underscore the transformative potential of multi-enzyme, one-bath systems in textile wet processing, moving toward a model of process consolidation, resource efficiency, and eco-innovation.

#### 2.6.2. Immobilised Enzyme Systems

A key limitation in the industrial application of enzymatic treatments is the uncontrolled activity of free enzymes, which can compromise fibre integrity. Additionally, the inability to easily reuse enzymes increases operation costs, hindering broader adoption in textile manufacturing. Enzyme immobilisation has emerged as a promising strategy to overcome these challenges by restricting enzymatic activity to the fibre surface and enabling multiple reuse cycles.

During enzyme-assisted finishing treatments, excessive cellulase activity can compromise fibre strength by entering deep into the fibre and degrading the internal structure of the fabric [12]. To address this, cellulase immobilisation on calcium beads was explored. Sankarraj et al. [43] demonstrated that cotton treated with cellulases immobilised in calcium alginate beads exhibited lower crystallinity index compared to free enzymes, indicating limited action on hydrolysis. In a subsequent study, the same research group improved both whiteness and tensile strength when using immobilised enzymes, compared to their free counterparts [44]. Enzyme reusability was also demonstrated in wool scouring, where lipase immobilised on sericin-based discs retained effectiveness across five treatment cycles [45]. However, testing temperatures and durations (room temperature for 12–24 h) were not aligned with the industrial reality, indicating the need for further optimisation to achieve scalability. In another study, amylase, pectinase and glucose-oxidase were covalently bonded to polymeric supports to perform desizing, scouring and bleaching of cotton [46]. Glucose oxidase produced H_2_O_2_ in situ during bleaching by oxidising added glucose. The immobilised enzymes were successfully reused across three cycles, maintaining whiteness index and acceptable colour differences, thus demonstrating operational durability.

Commercially, enzyme immobilisation is also gaining attraction, with companies such as Creative Enzymes^®^ (Shirley, NY, USA) offering multiple enzyme immobilisation techniques that can be tailored to client-specific textile applications.

Overall, these findings highlight enzyme immobilisation as a promising strategy to reduce high costs of individual enzyme applications by enabling enzyme reuse and improving fabric properties through more controlled and efficient action, while also contributing to process sustainability through reusability.

## 3. Biopolymers

Polymers are large molecules composed of repeating units known as monomers, which are chemically bonded together to form long chains. Each monomer is a small molecule capable of linking with others through polymerisation reactions. Polymers can be classified based on their origin into synthetic polymers, typically built from artificial monomers derived from petroleum-based feedstocks, and biopolymers, which are made from natural monomer sources such as plants, animals or microorganisms [47]. As already mentioned, in textile wet processing, synthetic auxiliaries pose significant risks to the environment and human health [3]. In this context, biopolymers have emerged as promising alternatives due to their biodegradability, renewability, and lower environmental impact [48].

Biopolymers include naturally occurring macromolecules such as proteins (polymers of amino acids), polysaccharides (polymers of sugars), nucleic acids (polymers of nucleotides), and some lipids that exhibit polymeric structures [48]. While many biopolymers are directly extracted from biological matter such as starch and chitosan, others are produced via chemical or microbial synthesis using bio-based feedstocks. For instance, polylactic acid (PLA) is a biopolymer derived from plant-based sugars that undergo fermentation followed by chemical polymerisation [49]. Although chemically synthesised, PLA is commonly classified as a synthetic biopolymer or bio-polyester due to its renewable biological origin.

A wide range of biopolymers is currently being explored in textile wet processing, particularly proteins and polysaccharides. These materials can be employed during pre-treatment processes for fibre modification, such as cationisation, to improve dye uptake and fixation. In finishing stages, they confer functionalities such as flame retardancy, antimicrobial, antioxidant, and ultraviolet (UV) radiation protection, while also contributing to environmental sustainability [50]. Table 2 summarises the main biopolymers applied in textile processes, with emphasis on their classification, chemical structure, processing steps where they can be applied and major function.

### 3.1. Pre-Treatment

Following enzymatic strategies, biopolymers have emerged as a promising class of alternatives for fibre pre-treatment in textile wet processing. These naturally derived macromolecules offer multifunctional properties that go beyond simple substitution, as they can modify fibre surfaces, enhance dye uptake, and improve compatibility with both synthetic and natural dyeing agents.

Among them, polysaccharide-based polymers such as chitosan have shown significant potential in cationisation of cellulosic fibres, offering a more sustainable alternative to synthetic agents. Their ability to introduce positive charges onto fibres’ surface facilitates the interaction with negatively charged dye molecules, thereby improving dye fixation and overall performance. Chitosan is obtained through the partial deacetylation of chitin, a naturally occurring polysaccharide abundantly present in the exoskeletons of crustaceans, insects and fungi [51]. This chemical modification removes acetyl groups, exposing free amino groups that become protonated under acidic conditions. As a result, chitosan acquires a positive charge, enabling it to interact with both the negatively charged surfaces of textile fibres, particularly cotton, as well as with anionic dyes and pigments [52]. These cationic properties make chitosan a sustainable and effective alternative to synthetic cationising agents. Furthermore, as chitosan is typically sourced from crustacean shell waste, its use aligns with circular economy principles and promotes waste valorisation. Several studies have demonstrated chitosan’s effectiveness in improving dye uptake and colour fastness properties. For instance, cotton pre-treated with a 4% chitosan solution improved dye absorption, exhibited higher K/S values and enhanced washing and light fastness when dyed with reactive dyes, compared to untreated samples [53]. Because natural dyes often suffer from limited uptake and poor fastness, chitosan has proven particularly effective in overcoming these challenges by enhancing dye absorption and improving colour durability. Cotton, wool, and silk fabrics pre-treated with chitosan and dyed with beetroot waste exhibited significantly increased K/S values, which was attributed to the electrostatic attraction between the protonated amino groups on chitosan and the anionic dye molecules [54]. Similar improvements in dye uptake, K/S and colour fastness have been reported using peanut shell on linen [55] and aronia berry on cotton [56]. Moreover, chitosan has been effectively used as a biomordant during cotton dyeing with turmeric root and onion dyes, achieving dye fixation and colour fastness comparable to conventional metal mordants [57,58]. Collectively, these findings confirm chitosan’s versatility and efficacy as a sustainable pre-treatment agent capable of improving dyeability while reducing environmental impact.

Beyond polysaccharides, protein-based biopolymers such as sericin and gelatine have also been effectively applied in fibre modification. Sericin, a silk protein, is rich in hydroxyl and carbonyl groups, which facilitate bonding with fibre surfaces. Pre-treating wool with 5% sericin prior to synthetic dyeing led to significantly increased K/S values when compared to the untreated fabrics and resulted in excellent washing fastness results [59]. Gelatine, a protein derived from collagen-rich fleshing waste of collagen via thermal hydrolysis, was used to cationise cotton for salt- and alkali-free dyeing. The treated fabrics resulted in higher K/S values and comparable colour fastness to the conventional method, and reduced TDS in the dyeing effluent [60]. However, one limitation is gelatine’s sensitivity to high temperatures, which may degrade its structure and compromise performance.

Biopolymer-based pre-treatments offer a sustainable and multifunctional alternative to conventional fibre modification auxiliaries. Polysaccharides such as chitosan not only enhance dye uptake and colour fastness but also promote circularity by repurposing crustacean shell waste. Similarly, protein-based biopolymers such as sericin and gelatine improve colour performance across various fibres, despite some limitations on thermal sensitivity. Overall, these materials show strong potential to support low-impact textile processing systems.

### 3.2. Printing

The printing of cellulosic fabrics typically involves the application of a printing paste, in conventional processes, or a fabric pre-treatment followed by printing, in digital processes. In both approaches, incorporating a polymer as a thickener is essential to carry the fixing agent and inhibit the migration of dye molecules during the printing, drying, and steaming steps [61,62].

Currently, sodium alginate, a biopolymer derived from brown algae, is the most used thickener for cotton printing due to its excellent solubility and stability. However, to expand applications to other fibres, there is a need to explore alternative biopolymers to be used as thickeners. These alternatives include natural gums, starch, and pectin.

Natural gums are polysaccharides primarily obtained from plants and microorganisms and can be classified according to their source as: (i) plant exudate gums; (ii) seed or root gums; (iii) microbial gums; and (iv) marine gums [63]. These biopolymers have been mainly explored as thickening agents, but some authors also studied their potential use in the pre-treatment for digital printing, as urea substitutes. The traditional digital printing pre-treatment formulations contain urea, which facilitates fibre swelling but is released into wastewater during washing, contributing to environmental pollution. Carboxymethyl guar gum (CMG), a seed gum extracted from *Cyamopsis tetragonolobus*, has been studied for its potential as a thickener and swelling agent. In a study performed by Khajeh Mehrizi et al. [64], modified CMG was evaluated as a thickener for printing cotton fabrics with reactive dyes, and its performance was compared to that of sodium alginate. The results revealed higher K/S and no significant effect on colour fastness. Zhang et al. [65] explored the use of CMG as both thickener and urea substitute in the inkjet printing pre-treatment formulation for hemp fabrics. As results, the CMG pre-treatment significantly improved K/S values and resulted in good colour fastness properties, with the prints exhibiting better colour uniformity and sharpness. Xanthan Gum, a microbial gum mostly produced by *Xanthomonas campestris* NRRL B-1459 strain, has also gained significant relevance in industrial applications. Hassabo et al. [66] studied the use of chemically modified xanthan gum as a thickening agent for inkjet printing on cotton, silk, and polyester fabrics using reactive dyes. Compared to native xanthan gum, the use of modified xanthan gum was able to increase the pre-treatment pick-up, colour yield, dye penetration, and outline sharpness.

Starch, a polysaccharide found mainly in plant sources such as corn, potatoes and various vegetables, was also successfully applied in printing processes. Mongkholrattanasit et al. [67] explored the use of a novel thickening agent derived from modified starch extracted from wild taro corms in the screen-printing of cotton fabrics with natural indigo dye. The results demonstrated good to very good colour fastness to washing, water, and perspiration. However, colour fastness to light and rubbing were rated as good and satisfactory, respectively. Vastrad et al. [68] explored the application of starch derived from mango kernels as a natural thickening agent for printing on cotton fabrics. Results showed that mango kernel starch has comparable performance to synthetic thickeners.

Another alternative biopolymer suitable for printing could be pectin, commonly found in the cell walls of all higher plants. Ebrahim et al. [62] studied the application of pectin extracted from orange and pomegranate peels as thickeners for printing pastes to be applied on cotton, polyester, polyacrylic and wool fabrics. The results revealed excellent washing fastness and strong performance in both dry and wet rubbing fastness. Additionally, these samples exhibited improved light and perspiration fastness when compared to sodium alginate samples. Despite the promising results, pectin application as a thickener is still limited due to its high costs.

The transition toward more sustainable textile printing methods has driven interest in biopolymer-based thickeners as alternatives to conventional materials such as sodium alginate. Among these, carboxymethyl guar gum, xanthan gum, starch, and pectin have shown potential to enhance K/S, colour fastness, and print quality while reducing environmental impact. Although promising, further research is needed to refine formulations, enhance durability, and support large-scale application. These bio-based thickeners offer a practical path toward greener and more responsible printing practices in the textile industry.

### 3.3. Finishing

Beyond their role in fibre pre-treatment and printing technologies improvement, biopolymers have also shown significant promise in textile functionalisation, where they contribute to value-added properties such as antimicrobial activity, flame retardancy, UV protection, wrinkle recovery, and antioxidant performance. Functional finishing using biopolymers aligns with the broader movement toward sustainable chemistry, as these macromolecules are often bio-based, biodegradable, and derived from renewable resources or industrial by-products. Their chemical versatility—offering hydroxyl, amino, carboxyl, or phosphate groups—enables them to serve as active agents or carriers for functional compounds, either through direct bonding to textile surfaces or as part of encapsulated or layered systems [69,70].

Chitosan, for example, continues to attract attention not only for its cationic properties but also for its inherent antimicrobial activity. In a study performed by Mosaad et al. [71], chitosan nanoparticles were applied to cotton fabric, significantly enhancing its antimicrobial performance. The study demonstrated that while unmodified chitosan was more effective against the Gram-positive bacteria *S. aureus* and *Bacillus subtilis*, chitosan nanoparticles extended this activity to Gram-negative bacteria such as *Escherichia coli* and *Proteus*. Antifungal efficacy against species such as *Aspergillus niger* and *Candida albicans* was also improved. The authors also found that the incorporation of acid dyes into the chitosan matrix further enhanced the overall antimicrobial effect, suggesting a possible synergistic interaction between the dye molecules and the bio-based matrix might have occurred.

Starch-based nanomaterials have also been explored as sustainable alternatives to conventional wrinkle- and shrinkage-resistant finishes, owing to their high surface area and distinct properties compared to native starch granules, which enable stronger fibre interactions and more uniform distribution. In cellulosic fabrics, wrinkling in the moist state is primarily due to the disruption of inter-chain hydrogen bonding, which leads to dimensional changes upon drying. Conventional synthetic finishes form covalent cross-links to address this, but often at the expense of handle, breathability, and environmental safety. A recent study addressed this by using starch nanoparticles to treat cotton fabric, resulting in a significant increase in wrinkle recovery angles in both the warp and weft directions, indicating better elastic recovery from creasing [72]. The treatment also led to an increase in fabric stiffness due to the formation of a surface film. However, after ten washing cycles, both stiffness and wrinkle recovery angles decreased, returning close to untreated values, suggesting the need for further improvements in durability. Nevertheless, this approach presents a bio-based alternative that avoids inorganic and non-renewable matrixes.

Lignin, a polyphenolic biopolymer available as a by-product of the paper and bioethanol industries, offers multifunctional properties for textiles. Nanolignin was applied to cotton and linen fabrics, imparting antimicrobial, anti-UV and antioxidant properties [73]. The treated fabrics demonstrated significant antibacterial activity, evidenced by inhibition zones of up to 18 mm for *S. aureus* and up to 12 mm for *Klebsiella pneumoniae*, and excellent UV protection factor (UPF) values. However, durability declined after washing, indicating leaching of nanolignin. Another study used ultrasound-assisted lignin application on ramie fabric, achieving strong to exceptional antimicrobial activity against *Cutibacterium acnes*, *S. aureus*, *Staphylococcus epidermidis* and *B. subtilis* [70]. Additionally, sodium lignin sulfonate applied to cotton at 30% concentration significantly improved flame retardancy and UPF values from good to excellent, showcasing lignin’s multifunctionality [74].

Casein, a phosphoprotein derived from milk, typically recovered as a by-product from the dairy or cheese industry, is also being investigated for its flame-retardant properties. In a study performed by Faheem et al. [75], casein was applied to cotton fabric under acidic conditions, where protonation allowed better electrostatic binding to the fibre surface. While flame resistance was achieved, durability was limited due to casein’s solubility and susceptibility to UV degradation. Additionally, high viscosity at elevated concentrations negatively affected the physiological comfort of the fabrics, suggesting that moderate application levels are necessary for functional textiles with balanced performance.

Gelatine has also been explored for flame-retardant applications via various strategies. One method involved a six-bilayer layer-by-layer coating of gelatine and ammonium polyphosphate on cotton fabric, reducing the flame damage length from 300 mm to 86 mm [69]. However, washing durability remained a limitation due to the solubility of both components. Other studies extended this approach to synthetic fabrics such as polyester and polypropylene, using gelatine, diammonium hydrogen phosphate, and silica gel in layer-by-layer assemblies, achieving flame retardancy times of 3 to 5 s with limited but acceptable mechanical degradation [76]. A further innovation involved phytic acid–gelatine coatings for polyester fabrics, achieving sustainable flame resistance with renewable phosphorus sources [77]. Finally, chitosan–gelatine microcapsules have also been developed to deliver bioactive essential oils, providing multifunctional finishes such as mosquito repellency, antibacterial activity, fragrance release, and antioxidant effects to linen fabrics, combining protection and sensory enhancement within a single biopolymer-based delivery system [78,79].

Biopolymers offer a sustainable and multifunctional approach to textile finishing, contributing to properties such as antimicrobial activity, flame retardancy, UV protection, and wrinkle resistance. Derived from renewable or waste sources, materials like chitosan, starch, lignin, casein, and gelatine not only reduce environmental impact but also enhance fabric performance. Despite challenges with durability and scalability, ongoing research continues to improve their application. Overall, biopolymers represent a promising shift toward greener, safer, and more innovative solutions in textile processing.

## 4. Bio-Based Colourants and Functional Agents

In addition to the previously mentioned compounds—such as enzymes and biopolymers—there is a wide range of other natural substances with significant potential in textile wet processes, particularly in dyeing and functionalisation. These include bioactive compounds derived from plants, agrowaste, and microbial metabolites, among others. Such naturally sourced materials offer sustainable and multifunctional alternatives to conventional chemicals, contributing to the development of more eco-friendly and innovative textile treatments. The following sections explore the potential of these compounds in natural dyeing and functionalisation processes.

### 4.1. Natural Dyeing

Natural dyes have been used for thousands of years, with indigo among the oldest recorded examples, known for over 5000 years [80]. Today, however, synthetic dyes have largely replaced natural dyes due to their broader colour range, consistent quality, lower cost, and superior fastness properties [81]. Despite these advantages, synthetic dyes pose significant ecological and health concerns. Derived from petrochemical compounds, their production can release toxic gases, and their use contributes to pollution of aquatic ecosystems and soil, disrupting the food chain and ultimately affecting human health [82]. These concerns have led to a renewed interest in natural dyes and the development of improved application methods.

Natural dyes are sourced from a wide range of materials, including earth minerals, insects, shellfish, various plant parts, agrowaste and microorganisms [80,83]. However, their successful application in textiles has had some challenges. A key limitation is their low affinity for textile fibres, which can result in poor colour uptake and weak fastness properties. This is particularly problematic as durability and colour consistency are critical in today’s standards.

To address this, mordants are used to improve natural dye fixation on textile fibres and enhance their fastness properties. Mordants can also help produce a broader shade range from a single dye source by altering dye-fibre interactions [84]. Their application can be done before dyeing (pre-mordanting), during dyeing (meta-mordanting), or after dyeing (post-mordanting). Pre-mordanting is the most widely used method, frequently producing better results, as the fabric is prepared with available binding sites before dye application [85]. However, a study by Rahman et al. [57] demonstrated that meta-mordanting only when the dyebath reaches the target temperature can outperform pre-mordanting in terms of fastness and resource savings. The effectiveness of each method depends on the type of mordant, dye, and fibre involved, so a universal technique remains elusive.

Traditionally, mordants are metallic compounds that help bind dyes to fabrics. Common metal salts used include aluminium (potash alum), iron (ferrous sulphate), copper (copper sulphate), tin (stannous chloride), and chromium (potassium dichromate) [85]. However, concerns over toxicity and environmental impact have led to research of more appropriate alternatives. Metal mordants may cause skin and respiratory irritation, affect internal organs, and pose carcinogenic risks, as in the case of chromium salts [86]. Moreover, only a small fraction of these salts binds to the fabric, with the remainder being washed out and discharged as effluent, contaminating water and soil ecosystems. As a more sustainable alternative, bio-mordants derived from natural sources such as plant extracts [87], and chitosan [57] are being investigated. These substances offer a safer and more ecofriendly way to fix natural dyes without compromising performance or harming the environment.

Given the growing motivation to replace synthetic dyes with natural alternatives and the importance of effective mordanting techniques, the following sections explore plant-, agrowaste- and microbial-based dye sources, as well as natural mordanting methods. Each of these sources presents distinct characteristics, challenges, and opportunities for sustainable textile colouration. In addition to colouration, many natural dyes possess functional properties such as antimicrobial, antioxidant, or UV-blocking effects, that align with the growing demand for multifunctional textiles. These attributes will also be discussed, alongside each dye source, when relevant.

#### 4.1.1. Plant and Agrowaste-Based Colouring Agents

Plants are highly versatile sources of natural dyes and have historically served as the primary origin of most natural colourants. Dye compounds can be extracted from various plant parts, including leaves, stems, flowers, fruits, and roots. A prominent example is indigo derived from the leaves of *Indigofera tinctoria*, one of the oldest known natural dyes, that continues to be used today, especially for denim dyeing, as a natural alternative to synthetic indigo [85].

Among the various plant parts, leaves are particularly attractive for dye extraction due to their availability, ease of regeneration, and limited plant damage. For instance, leaves of *Mimusops elengi* Linn were successfully applied on cotton and silk fabrics using aloe vera as a biomordant, resulting in good K/S and good to excellent colour fastness. Silk, being protein-based, demonstrated better results than cotton due to its greater affinity for natural dyes [88]. Similarly, red sepals from *Mussaenda erythrophylla* were used to dye silk, cotton and polyester fabrics, with lemon juice as a biomordant. This method achieved higher K/S compared to conventional metal mordants and produced good to very good colour fastness for silk fabrics [89].

Apart from leaves, other plant parts can also serve as sources of natural dyes. For example, in wool dyeing, roots of *Rubia tinctorum* (madder) and flowers of *Reseda luteola* were used along with myrobalan (yellow and black varieties) as a natural mordant in a meta-mordanting process. This approach not only produced reddish and brown shades with good colour fastness values but also improved sustainability by combining mordanting and dyeing steps, thus reducing water, energy and chemical consumption [87]. For cotton, curcumin was applied using chitosan as a natural mordant, added after the dyebath reached its target temperature. This method resulted in K/S values comparable to those achieved with conventional metal mordants, offering a more sustainable alternative [57].

Natural mordants have proven effective in enhancing colour fastness, emerging as sustainable alternatives capable of matching the performance of metal salts. Despite these advantages, a significant challenge remains as some plant-derived dyes, as those from rhizomes (e.g., curcumin) or roots (e.g., madder), often involve destructive harvesting that limits sustainability. While leaves and flowers are more renewable, they still require regrowth periods and are subject to seasonal availability. Additionally, scaling up plant-based dye production for industrial applications may demand extensive land use and resource input, which could undermine their environmental benefits.

To address these challenges, a compelling alternative is the use of agrowaste as dye sources. This approach not only provides a consistent and lower-cost supply of dye precursors but also offers a sustainable solution for waste management. Agrowaste-derived dyes align with circular economy principles by valorising agricultural and food-processing by-products.

For example, pistachio soft and hard shells yielded light brown shades on cotton fabrics when meta-mordanted with a corn powder [90]. This process produced very good to excellent colour fastness to washing, rubbing, and perspiration. Light fastness was limited; however, results were comparable to those obtained using metal mordants. Walnut green husks, rich in bioactive compounds, were used to dye cotton fabrics without salts or mordants, yielding brown, orange, and grey shades. While this method produced fair colour fastness, the 12-h dyeing process at room temperature, is not considered feasible for large-scale textile production [91].

Pomegranate by-products have emerged as another promising waste dye source. Peel extracts produced grey and brown shades on silk using Chinese quince (*Chaenomeles speciosa*) fruit as a biomordant, achieving higher K/S values and good to very good fastness compared to metal mordants [92]. When combined with gum rosin as a natural fixing agent, excellent fastness to washing, perspiration, and wet rubbing was attained. Fallen pomegranate leaves were used to dye wool without mordants, achieving very good colour fastness [93]. However, the high dye concentration used (99.79% owf) poses challenges for industrial scalability.

Beetroot waste, particularly peels, was used to extract betalains, which are bioactive compounds with vivid colouring potential. In the study performed by Al-Amir et al. [54], chitosan pre-treatment of cotton, wool and silk improved dye uptake and yielded very good to excellent colour fastness to washing, perspiration and light, with wool and silk showing better performance. Another study demonstrated that protonated wool, treated with acetic acid, dyed with beetroot betalains exhibited deep red shades with excellent colour fastness properties [94].

Grape pomace was also explored as a natural dye source for wool and cationised cotton, resulting in brown shades [95]. However, the study lacked essential data on colour fastness, limiting its evaluation.

These results demonstrate the broad potential of both plant- and agrowaste-based dyes across various fabric substrates and mordant systems. Given the volume and diversity of sources, a comparative overview is useful to highlight key aspects such as colour outcomes, substrate compatibility, mordants used, and overall fastness performance. Table 3 presents a consolidated summary of the plant- and agrowaste-based dye sources discussed so far and additional studies.

While the use of plant- and agrowaste-based dyes aligns with sustainability goals, certain practices and process conditions hinder their industrial-scale adoption. For example, the addition of a mordanting step frequently involves an additional high-temperature step, further increasing energy costs of an already high-temperature process. Conversely, sustainable dyeing approaches that operate at room temperature often require long dyeing durations, which are not viable for industrial applications. A further constraint is the high concentration of natural dye often required to achieve desirable shades and performance. This raises concerns about the scalability of raw material supply and the consistency of natural dye sources. Furthermore, natural dyes are often associated with reduced durability, particularly limited washing and light fastness, which constrains their long-term use in demanding textile applications. Additionally, one of the most frequently mentioned drawbacks of natural dyeing is its current cost, which tends to be higher than that of synthetic dyeing [101]. This cost disparity is primarily due to relatively low supply chains and limited demand for natural dyes in industrial applications. As with enzymes, increased adoption could lead to more competitive pricing over time.

Another important aspect is consumer safety. Some natural dye extracts may contain allergenic or irritant compounds, which may cause skin reactions. Systematic toxicological and dermatological testing is therefore essential to support regulatory approval and consumer confidence.

Several companies are already selling natural dyes, such as Natural dyes (Istanbul, Turkey), Sodhani Biotech (Jaipur, India) and Indidye (Shanghai, China). Some companies such as Nature Coatings (Las Vegas, NV, USA) are already working with waste-based sources, specifically wood waste to produce BioBlack TX. Despite some companies already selling natural dyes, natural dyes are still emerging as a serious alternative to synthetic dyes, and far to be considered an alternative to synthetic dyes by manufacturers.

#### 4.1.2. Microbial-Based Colouring Agents

When exposed to stress conditions, microorganisms produce secondary metabolites that display a diverse range of colourful molecules, including carotenoids, flavonoids, quinones, indigo, melanins, phenazines, violacein and prodigiocins [102]. These metabolites are classified as bio-pigments due to their capacity to impart colour to textile fibres [103,104]. However, from a textile-scientific perspective, they exhibit a behaviour more characteristic of dyes than pigments. Although they are not soluble in water, it has been shown that they are chemically bonded to textile materials, forming bonds with functional groups on fibres, indicating a dye-like interaction rather than simple surface adhesion [104,105].

Microbial pigments represent a promising source of natural dyes, capable of colouring a wide range of textile materials with intense colours that are non-toxic and safe for humans [105]. Compared to plant-based and synthetic dyes, microbial pigments provide numerous benefits. Microorganisms enable continuous production unaffected by seasonal variations or arable land availability, are easily isolated and propagated, have higher productivity, and enable versatility in the production process. Additionally, they can be genetically manipulated to optimise production or to create different colours, and the pigment extraction process is straightforward [103,106]. From an economic standpoint, microbial pigments also attract interest due to their low-cost production, fast growth, easy regulation of microbial cell factories for high production yields, and the potential of using different waste streams as a cheap culture medium [104]. These advantages contribute to reduced water and energy consumption in the dyeing process, which result in lower CO_2_ emissions and decreased wastewater discharge [107].

While fungi and microalgae are also capable of producing bio-pigments, bacteria are generally preferred due to their simple culturing and pigment extraction techniques, high scalability potential, and faster growth rates [104,105]. Among them, the most significantly studied genera include *Serratia, Streptomyces*, and *Pseudomonas*, which can be readily isolated from diverse environmental sources such as soil, water, plants, and insects [105,108].

Microbial pigment production begins with the selection of a suitable microorganism or the development of a genetically engineered strain. The selected microbe is then cultivated through fermentation using sugars and other feedstocks as nutrient sources. After fermentation, the microbial pigments are extracted and can be applied in the conventional dyeing process [109].

Although still in its early stages, the use of microbial pigments in the textile industry is gaining attention. Several studies have explored their potential, and a few companies have already begun commercialising these dyes.

Regarding research studies, Chadni et al. [110] isolated a strain of *Talaromyces verruculosus* that produces a red pigment, which was extracted and applied with a mordant to dye a cotton fabric. The results showed satisfactory dyeing performance, and a good washing resistance. Sengupta et al. [111] isolated a blue-green pigment from soil bacteria and explored its feasibility as a sustainable dye in textiles and paper. The results showed excellent dye uptake, good fastness properties, and compatibility with different fibres, demonstrating its viability as an effective and safe dye. Kıcık et al. [102] studied the dyeing of an 100% cotton fabric using three bacterial pigments (pink, blue, and brown) across six different dyeing processes. Results showed that each dyestuff reacted in a different way with a different process. However, all dyed fabrics exhibited good fastness properties.

In terms of companies developing microbial-based pigments, Colorifix (Norwich, England), Pili (Toulouse, France), Huue (Berkeley, CA, USA), Vienna Textile Lab (Vienna, Austria), and KBCols Science (Pune, India) are pioneering start-ups leading the commercialisation of microbial pigments for the textile industry [107]. Colorifix, Pili and Huue rely on genetically modified organisms (GMO) for pigment production, while KBCols Sciences and Vienna Textile Lab use naturally pigmented microbes sourced from the soil, water and air [109]. Furthermore, Pili and Huue focus on indigo, whereas Colorifix, KBCols Sciences and Vienna Textile Lab offer a broader colour palette [109]. An overview of each start-up can be found in Table 4.

Microbial dyeing attracted significant research interest in the early 2000s, emerging as a promising alternative to synthetic dyes due to its environmental benefits. However, as the technology began to transition toward industrial-scale applications, academic and experimental research in this area has noticeably declined. Today, there is a relative scarcity of recent scientific studies focused on the experimental development of microbial dyeing techniques.

### 4.2. Functional Finishing

Beyond their primary role in colouration, natural dyes can impart value-added functionalities to textiles, notably antimicrobial activity and UV protection. These properties are influenced by both the dye source and the mordanting agent used. As already discussed, metal mordants have traditionally been used to enhance such effects due to their inherent activity. For example, when used alone, coffee ground dye applied to surgical facemasks exhibited poor perspiration fastness and only antibacterial activity against *S. aureus*. However, when combined with a metal mordant, both properties improved significantly, expanding antimicrobial efficacy to *K. pneumoniae* as well [112]. Similarly, broccoli-derived dye applied to cotton provided good antibacterial and UV-protection performance only in the presence of metal mordants [101]. Nevertheless, as previously discussed, environmental and health concerns associated with metal mordants have prompted increased attention toward natural and bio-based mordants. This review emphasises these alternatives, highlighting their potential to deliver comparable performance while aligning with sustainable development goals.

#### 4.2.1. Plant- and Agrowaste-Based Agents

Numerous studies show that natural dyes derived from plants and agrowaste can enhance functional properties in textiles. For instance, sweet potato leaf extract was applied to silk, wool, cotton, polyester and polyamide without the use of salts or mordants. The dyed fabrics retained strong antibacterial activity against *E. coli* and *S. aureus* after thirty washing cycles (>83.8%) along with very good to excellent fastness across all substrates. Dyeing with this extract also enhanced UV protection, particularly for wool, which naturally presents UV protection, and elevated polyester and polyamide UPF from poor to very good, a value that remained stable for at least one month. Additionally, the residual dye bath was successfully reused up to five times, each producing lighter, yet acceptable shades, demonstrating the potential for resource-efficient dyeing [113].

Both *Acacia auriculiformis* bark and *Cinnamomum camphora* leaves were used to dye wool in brown shades, with studies reporting similar outcomes. The results showed strong antibacterial activity against *E. coli*, *S. aureus*, and *C. albicans*, and excellent UV protection, with UPF values exceeding 80 in some treatments, along with excellent washing and perspiration fastness [114,115]. Similarly, natural waste dyes from madder root, chamomile, pomegranate peels and apple tree bark achieved high UPF values and very good to excellent fastness properties, including excellent light fastness for pomegranate, all achieved without requiring metal mordants [116].

Silk fabrics biomordanted with leaves from *Mucuna pruriens* L. and *Justicia carnea* L. and dyed with mango leaves produced brown shades with UPF rating above 50, remaining above 45 after three exposures to light, along with excellent fastness to washing and perspiration, and very good to excellent light fastness. Notably, in this study, metal mordants were less effective than their natural counterparts [117].

Functionalisation with natural dyes was also successfully obtained in cotton. Fabrics dyed with marigold flowers and pomegranate peels displayed excellent UV protection and moderate light fastness without requiring mordants. Additionally, fabrics dyed with turmeric rhizome, onion peel and marigold flowers demonstrated over 50% mosquito repellency [118]. Cotton biomordanted with chitosan and dyed with onion skins achieved over 97% antibacterial efficacy against *E. coli* and *S. aureus*, retaining more than 80% effectiveness after twenty washing cycles, alongside excellent UPF values throughout [58]. Furthermore, cotton fabrics dyed with pomegranate rind and onion peel extracts exhibited very good to excellent washing fastness, up to 99.9% bacterial reduction, and over 90% antioxidant activity [119].

These findings collectively highlight the dual role of natural dyes in both textile colouration and functional enhancement, particularly when combined with eco-friendly mordants or applied in synergistic plant combinations. A summary of the reviewed studies, including dye source, mordant, textile substrate, fastness performance, and functional properties, is presented in Table 5.

Overall, research indicates that silk and wool generally exhibit better dye uptake and fastness in a wide range of plant- and agrowaste-based natural dyes. In contrast, cotton often requires stronger mordanting, higher dye concentration or the use of advanced techniques, such as ultrasound assistance, to achieve comparable results. Given that natural dyeing is still emerging as a viable alternative to synthetic dyes, most studies have focused on natural fibres. However, considering the continued prevalence of synthetic fibres in the textile industry, research exploring the application of natural dyes to synthetic substrates is growing, although limited when compared to those involving natural fibres. Similarly, functionalisation using plant- and agrowaste-derived agents faces comparable challenges, including the need for additional processing steps, longer treatment times, higher material demand, and higher costs, all of which currently limit large-scale adoption despite promising laboratory results.

#### 4.2.2. Microbial-Based Agents

As the plant- and agrowaste-based agents, microbial metabolites not only provide colour but also possess various functional properties, such as antimicrobial, anticancer, antioxidant, and UV-protective activities. These additional functionalities can improve textiles quality by offering benefits beyond colouration [105]. Simultaneous dyeing and finishing in a single bath helps save energy, time, and labour, thus significantly reducing production costs and resource consumption.

The antimicrobial activity of microbial dyes has been extensively studied in textile applications. However, other functional properties, such as antioxidant or anticancer effects, while well established in food and pharmaceutical fields, remain largely unexplored in textiles.

Venil et al. [127] explored a bacterium isolated from the enteric gut of insects, as a sustainable natural pigment for textile dyeing. The results displayed a strong affinity of the pigment for natural fibres and satisfactory fastness properties. Moreover, the dyed fabrics and yarns showed notable antibacterial activity, producing clear zones of inhibition against pathogenic bacteria including *B. subtilis*, *E. coli*, and *Pseudomonas aeruginosa*. Darwesh et al. [128] combined microbial pigments from *Streptomyces torulosus* with silver nanoparticles to dye textiles and add antimicrobial and anticancer properties. The resulting fabrics, wool and polyamide, showed a good to very good colour fastness, and the combination with silver nanoparticles enhanced antimicrobial activity against *S. aureus*, *Bacillus cereus*, *E. coli*, *Salmonella Typhi*, *C. albicans*, *Saccharomyces cerevisiae* and *A. niger*, and anticancer properties against A431 carcinoma cell line. Madhu et al. [129] investigated the use of a natural pigment derived from *Psilocybe zapotecorum* mushrooms to colour fabrics while imparting antibacterial functionalities. The results exhibited good colour fastness and clear antimicrobial activity against *S. aureus* and *E. coli*. Ren et al. [130] investigated the use of prodigiosin—a red pigment produced by *Serratia marcescens*—in cotton dyeing. The pigment was applied in the form of nanosuspensions of prodigiosin micelles, obtained via microbial fermentation. The treated cotton fabrics were successfully dyed and exhibited significant antibacterial properties against *S. aureus* and *E. coli*.

A summary of the reviewed studies, including the microorganism/pigment used and respective colour, textile substrate, fastness properties, and functional activity, is presented in Table 6.

Microbial metabolites represent a promising alternative by combining colouration with added functional properties. While antimicrobial applications have been the most extensively explored, recent studies demonstrate the potential of microbial pigments to deliver multifunctional properties when integrated into textile substrates. These findings highlight the dual role of microbial pigments in achieving both aesthetic and performance enhancements, while also contributing to more sustainable and cost-efficient production processes through simultaneous dyeing and finishing. However, further research is still needed to expand the application of non-antimicrobial functionalities, ensuring broader exploitation of microbial agents in the textile industry.

## 5. Conclusions and Future Directions

The transition toward sustainable textile wet processing is advancing rapidly through the integration of bio-based innovations, with enzymes, biopolymers, and natural compounds emerging as credible alternatives to conventional chemical auxiliaries.

Enzymes have proven particularly effective in pre-treatment processes, offering improved fabric performance while reducing environmental impact. However, their limited stability under harsh industrial conditions and relatively high costs continue to restrict widespread adoption. Recent progress in genetic engineering, extremophile enzyme discovery, and enzyme immobilisation offers promising solutions to overcome these barriers and align enzymatic systems more closely with industrial requirements.

Biopolymers such as chitosan, starch, lignin, casein, and protein derivatives provide additional opportunities to enhance textile functionality while supporting circular economy principles through the valorisation of agro- and food-industry by-products. Their role in fibre modification, dyeing, printing, and finishing underscores their versatility, although challenges regarding durability, reproducibility, and large-scale application remain to be resolved. Parallel advancements in formulation and processing methods will be essential to enable their broader industrial uptake.

Natural dyes and bioactive compounds derived from plants, agrowaste, and microbial sources represent another promising pathway toward greener textile treatments. While they provide significant environmental advantages, current limitations include scalability, cost competitiveness, process optimisation, and raw material consistency. Mordanting requirements, energy-intensive dyeing conditions, and the high quantities of natural dye needed for effective colouration further constrain their industrial integration. Nonetheless, growing commercial interest and early industrial applications suggest that these barriers may gradually be addressed. Importantly, future efforts must also include comprehensive toxicological and ecological assessments to ensure the safety and regulatory compliance of natural dyes derived from microorganisms, and agricultural residues or other waste streams.

Looking ahead, several key directions will shape the future of sustainable textile wet processing. First, continued research into extremophilic and genetically engineered enzymes, coupled with innovative immobilisation strategies, will be crucial for achieving economically viable and robust biocatalytic systems. Second, the optimisation of biopolymer-based auxiliaries, including blends and functionalised derivatives, can expand their industrial applications while improving performance and durability. Third, future research and industrial adoption should also consider bio-based surfactants, which can complement enzymes, biopolymers, and natural dyes by enhancing wetting and fibre penetration, thereby contributing to more sustainable and efficient textile wet processing methods. Finally, scaling natural dyeing processes will require not only technological improvements but also the establishment of resilient supply chains, standardisation of raw materials, and industry-wide collaborations. Equally important, future work must integrate process-level optimisation, including reduced bath ratios, efficient water and heat recovery, and better energy management, to ensure that bio-based alternatives deliver both chemical and thermodynamic sustainability.

While bio-based alternatives significantly reduce toxic chemical loads, their impact on solid release must also be considered. Treatments such as enzymatic finishing can increase the shedding of cellulosic fibrils, which contribute to suspended solids in effluents and may interact with residual bioactive compounds from natural dyeing, potentially influencing downstream treatment efficiency. Future studies should therefore evaluate both the chemical and particulate composition of wastewater, including bioactive substance content and fibril release, to ensure that bio-based processing achieves a substantial reduction in environmental impact.

Taken together, these innovations reflect a paradigm shift in textile wet processing, moving from reliance on synthetic, resource-intensive methods toward more sustainable, multifunctional, and circular solutions. While significant challenges remain, the integration of bio-based auxiliaries has the potential to transform the textile industry into a more environmentally responsible and technologically advanced sector.

## Figures and Tables

**Table 1:** Summary of the enzymes used in textile wet processing and respective function (from [9,10,11]).

Classification	Enzyme	Wet Processing Step	Function
Hydrolases	Amylase	Desizing	Removal of starch-based sizes
	Lipase	Desizing and scouring	Removal of fats and oils
	Pectinase	Scouring	Removal of non-cellulosic contents (e.g., pectin, waxes, protein, ashes and seed husk fragments)
	Cutinase	Scouring	Removal of cutin and waxes
	Protease	Scouring and finishing	Degumming of silk; modification of wool and silk
	Xylanase	Scouring and bleaching	Removal of residual hemicellulose
	Cellulase	Finishing	Removal of cellulosic fibres on the textile’s surface (fibrils)
Oxidoreductases	Catalase	Bleaching	Decomposition of residual H_2_O_2_ after bleaching
	Laccase	Bleaching and dyeing	Removal of indigo from denim fabrics and oxidation of dye precursors
	Peroxidase	Bleaching	Oxidation of dyes that are not bond covalently and removal of residual H_2_O_2_
	Glucose oxidase	Bleaching	Generation of H_2_O_2_

**Table 2:** Summary of the biopolymers used in textile wet processing and respective function [50].

Classification	Origin	Biopolymer	Structure	Wet Processing Step	Function
Polysaccharides	Animal	Chitosan	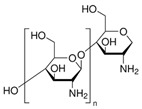	Pre-treatment and finishing	Dye uptake improvement Antimicrobial Flame retardancy Microencapsulation
	Plant	Alginate	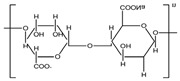	Printing	Thickener
	Plant	Starch	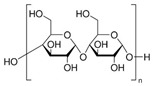	Printing and finishing	Thickener
	Plant	Natural gums	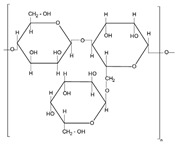	Printing	Thickener Swelling agent
Polyphenolics (not true polysaccharides)	Plant	Lignin	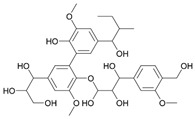	Pre-treatment and finishing	Antibacterial UV protection Antioxidant Flame retardancy
Proteins	Animal	Sericin	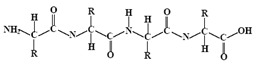	Pre-treatment	Dye uptake improvement
	Animal	Casein	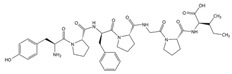	Finishing	Flame retardancy
	Animal	Gelatine	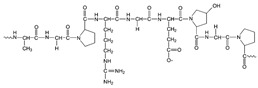	Pre-treatment and finishing	Dye uptake improvement Flame retardancy Microencapsulation

**Table 3:** Summary of plant- and agrowaste-based natural dyes, their resulting colours, used mordants, applied substrates, and corresponding colour fastness properties.

Dye Source	Colour	Mordant/ Application	Substrate	Colour Fastness	Ref
Plant-Based Dyes					
*Mimusops elengi* Linn leaves	N.a.	Aloe vera (pre-, meta- and post-mordanting)	Cotton (CO) Silk (SK)	CO/No mordant: W 5; R 4-5 CO/Aloe vera: W 5; R 5 SK/No mordant: W 5; R 5 SK/Aloe vera: W 5; R 5	[88]
*Mussaenda erythrophylla* red sepals	Peach	Lemon juice (pre-mordanting)	Silk (SK)	W 4; R 3; P 2-3; L 3-4	[89]
Madder	Reddish Brown	Yellow myrobalan (YM) Black myrobalan (BM) (pre- and a meta-mordanting)	Wool (WO)	20% madder/20% YM: W 3; R 4-5; L 4-5 10% madder/20% BM: W 4; R 4-5; L 3 10% reseda/10% YM: W 3-4; R 4-5; L 4 10% reseda/10% BM: W 3-4; R 4-5; L 3	[87]
Turmeric root	Yellow	Chitosan (meta-mordanting: added at the beginning or after reaching dyeing temperature)	Cotton (CO)	W 4-5	[57]
St. John’s wort Onion peel	Light brown Orange	Laccase-assisted colouration	Cotton (CO)	St. John’s: W 4; R 4; L 3 Onion: W 4-5; R 4; L 3	[21]
Agrowaste-Based Dyes					
Pistachio soft and hard shells	Light brown	Tannic acid (TA) Acorn powder (AP, pre- and meta-mordanting)	Cotton (CO)	Soft shells/TA: W 4-5; R 4-5; P 5; L 3 Hard shells/TA: W 5; R 4-5; P 5; L 3-4 Soft shells/AP: W 5; R 4-5; P 5; L 3 Hard shells/AP: W 5; R 4-5; P 5; L 3	[90]
Walnut green husks	Brown Orange Grey	No mordant No salt	Cotton (CO)	W 2; R 3-4	[91]
Onion peel	Brown	Banana peel Guava leaves (pre-, meta- and post-mordanting)	Jute/Cotton (CJ/CO)	Banana: W 3-4 Guava: W 3	[96]
Pomegranate peel	Grey Brown	Chinese quince fruit (pre-, meta- and post-mordanting)	Silk (SK)	W 3-4; R 4-5; P 4	[92]
Pomegranate fallen leaves	N.a.	No mordant	Wool (WO)	W 4-5; R 4; L 6-7	[93]
Beetroot waste	N.a.	Chitosan (pre-mordanting)	Cotton (CO) Wool (WO) Silk (SK)	CO: W 4; P 4-5; L 4-5 WO: W 4-5; P 5; L 5 SK: W 5; P 5; L 5	[54]
Beetroot peel	Red	Acetic acid (pre-mordanting)	Wool (WO)	W 5; R 4-5; L 5	[94]
Grape pomace	Brown	Cationisation (CO) No mordant (WO)	Cotton (CO) Wool (WO)	N.a.	[95]
Marigold flower	Yellow	Ultrasound-assisted	Cotton (CO)	W 3; R 4-5; L 7	[97]
Annato	Orange	No mordant	Recycled polyester (rPES)	W 5; R 5	[98]
Turmeric Madder Indigo Henna Catechu (Powder, Pd, and Liquid, Lq, forms)	Yellow Red Blue Green Brown	No mordant	Polyester (PES)	Turmeric Pd (5%): W 5; R 4-5; L 1 Turmeric Lq (5%): W 4; R 3-4; L 1-2 Madder Pd (10%): W 5; R 3-4; L 3 Madder Lq (10%): W 4-5; R 2; L 3 Indigo Pd (3%): W 4-5; R 3-4; L 3 Indigo Lq (3%): W 5; R 4; L 3-4 Henna Pd (5%): W 5; R 3-4; L 2 Henna Lq (5%): W 5; R 2-3; L 2 Catechu Pd (2.5%): W 5; R 3; L 1-2 Catechu Lq (2.5%): W 4; R 2; L 3-4	[99]
Saffron powder	Yellow	Pomegranate Turmeric (pre- and post-mordanting)	Polyamide (PA)	Pomegranate: W 4-5; P 4-5; L 5 Turmeric: W 4; P 4-5; L 4-5	[100]

Colour fastness (W-washing, R-rubbing, P-perspiration): (1) poor; (2) fair; (3) good; (4) very good; (5) excellent. Light fastness (L): (1) very poor; (2) poor; (3) fair; (4) moderate; (5) good; (6) very good; (7) excellent; (8) outstanding. N.a.—not available; Pd—powder; Lq-liquid; YM—yellow myrobalan; BM—black myrobalan; TA – tannic acid; AP – acorn powder; CO—cotton; SK—silk; WO—wool; CJ—jute; rPES—recycled polyester; PES—polyester; PA—polyamide.

**Table 4:** Commercial pigment profiles of Colorifix, Pili, Huue, Vienna Textile Labs, and KBCols Sciences, highlighting the microorganism used, the colours available, and suitable textile substrate.

Start-Up	Microorganism	Colours	Substrate
Colorifix	GMO	Diverse	Natural and Synthetic
Pili	GMO	Indigo	Cellulosic, proteic and blended fibres
Huue	GMO	Indigo	Cellulosic, proteic and blended fibres
Vienna Textile Lab	Bacteria and Fungi	Diverse	Natural and Synthetic
KBCols Sciences	Bacteria and Fungi	Diverse	Natural and Synthetic

**Table 5:** Summary of plant- and agrowaste-based functional agents used for textile functionalisation, highlighting the source, type of mordant, textile substrate used, fastness performance, and functional properties.

Dye Source	Mordant/ Application	Substrate	Colour Fastness	Functionalities	Ref
Sweet potato	No mordant No salt	Silk (SK) Wool (WO) Cotton (CO) Polyester (PES) Polyamide (PA)	N.a.	AM: >80% after 30 washing cycles (*E. coli* and *S. aureus*) UPF: WO, Excellent after 1 month; PA, PES, Very good after 1 month	[113]
*Acacia auriculiformis* bark	No mordant	Wool (WO) Silk (SK) Cotton (CO)	WO, SK: W 4-5; R 4-5; L 4-5 CO: W 4; R 4-5; L 3-4	AM: >90% (*E. coli* and *S. aureus*) UPF: WO, Excellent; SK, CO, Good	[114]
*Cinnamomum camphora* leaves	No mordant	Wool (WO)	W 4-5; L 3	AM: NaOH extract, >90% (*E. coli* and *S. aureus*), >85% (*C. albicans*); water extract, 65–80% (*E. coli*, *S. aureus* and *C. albicans*) UPF: Excellent	[115]
Pomegranate peel Chamomile Apple tree bark Madder roots	No mordant	Wool (WO)	Pomegranate: W 5; R 4; L 7 Chamomile: W 5; R 4; L 4 Apple: W 5; R 4-5; L 3-4 Madder: W 5; R 3-4; L 2-3	UPF: Excellent	[116]
Chinese tallow leaves	Chlorophyll extract (pre-mordanting)	Wool (WO)	No mordant: W 4-5; R 4 Chlorophyll: W 5; R 4-5	AM: No mordant, >95% (*B. subtilis*), >90%, (*P. aeruginosa*); Chlorophyll, >95% (*B. subtilis*), 70% (*P. aeruginosa*) UPF: Very good Aox: No mordant, 70%; chlorophyll, 50%	[120]
Mango leaves	*Mucuna pruriens* L. leaves *Justicia* *carnea* L. leaves (pre- and post-mordanting)	Silk (SK)	Mucuna/No microwave: W 5; R 5; L 7 Mucuna/Microwave: W 5; R 5; L 7-8 Justicia/No microwave: W 5; R 5; L 7-8 Justicia/Microwave: W 5; R 5; L 8	UPF: Excellent after 3 exposure periods Aox: >80% after 3 washing cycles	[117]
Henna leaves Turmeric rhizome Pomegranate peel Onion peel Marigold flowers Eucalyptus bark Acacia bark	No mordant	Cotton (CO)	Henna: W 4; R 4-5; L 5 Turmeric: W 3; R 4-5; L 5-6 Pomegranate: W 3; R 4-5; L 4 Onion: W 4; R 4-5; L 5 Marigold: W 3; R 4-5; L 4 Eucalyptus W 4; R 4-5; L 4 Acacia: W 4; R 4-5; L 4	AM: No activity (*E. coli* and *S. aureus*) UPF: Marigold, Pomegranate, Eucalyptus, Excellent; Henna, Acacia, Onion, Very good; Turmeric, Insufficient MR: Turmeric, Onion, Marigold, >50%	[118]
Onion peel	Chitosan (pre-mordanting)	Cotton (CO)	N.a.	AM: >95% (*E. coli* and *S. aureus*); >80% after 20 washing cycles UPF: Excellent after 20 washing cycles	[58]
Pomegranate rind Onion peel Turmeric root	No mordant	Cotton (CO)	Pomegranate: W 4-5; R 4-5 Onion: W 4-5; R 4-5 Turmeric: W 3-4; R 4	AM: Pomegranate, 99.9% (*S. aureus* and *K. pneumoniae*); Onion and Turmeric, 99.9% (*S. aureus*) UPF: Pomegranate, Very good; Onion, Good; turmeric, Insufficient Aox: Pomegranate, Onion, >90%	[119]
Sugarcane bagasse Wheat bran Rice husk	Seed coats tamarind (pre-, meta- and post-mordanting)	Cotton (CO)	Sugarcane/Pre-mordanting: W 4; L 2-3 Sugarcane/Post-mordanting: W 4; L 4 Wheat/Pre-mordanting: W 4; L 2-3 Wheat/Post-mordanting: W 3-4; L 4 Rice/Pre- mordanting: W 4; L 2-3 Rice/Post-mordanting: W 3-4; L 4	UPF: Rice/Pre-mordanting, Very good; Sugarcane/Pre-mordanting, Wheat/Pre-mordanting, Good; Post-mordanting, Insufficient	[121]
*Achillea millefolium* L.	Ultrasound-assisted	Cotton (CO)	N.a.	AM: Contact inhibition (*E. coli* and *S. aureus*)	[122]
Pomegranate peel	No mordant	Polyester (PES)	W 4; R 4; L 5	AM: 90% (*S. aureus*) UPF: Excellent Aox: 93.97%	[123]
Hawthorn fruits	Ultrasound-assisted	Polyamide (PA)	W 5; R 4; P 4-5; L 7	AM: 50% owf, 60% (*E. coli*) 47% (*S. aureus*); 100% owf, 91% (*E. coli*) 81% (*S. aureus*) Aox: 50% owf, 80%; 100% owf, >85%	[124]
Pomegranate peel	Gallnut (meta-mordanting)	Polyamide (PA)	W 3-4; R 4-5; L 2-3	AM: 60% (*E. coli*), 50% (*S. aureus*)	[125]

Colour fastness (W-washing, R-rubbing, P-perspiration): (1) poor; (2) fair; (3) good; (4) very good; (5) excellent. Light fastness (L): (1) very poor; (2) poor; (3) fair; (4) moderate; (5) good; (6) very good; (7) excellent; (8) outstanding. UPF (<15) poor; (15–24) good; (25–39) very good; (40–50, 50+) excellent [126]. N.a.—not available; SK—silk; WO—wool; CO—cotton; PES—polyester; PA—polyamide; AM—antimicrobial activity; UPF—UV protection factor; Aox—antioxidant activity; MR—mosquito repellency.

**Table 6:** Summary of microbial agents used for textile dyeing and functionalisation, highlighting the pigment or microorganism used, colour, textile substrate used, experimental conditions, fastness performance, and functional properties.

Microorganism /Pigment	Colour	Substrate	Colour Fastness	Functionalities	Ref
*Serratia marcescens*	Red	Acrylic (PAC)	W 5; R 5; P 5	N.a.	[131]
*Pseudomonas aeruginosa*	N.a.	Polyester (PES)	W 4-5; P 4-5	N.a.	[132]
*Streptomyces* sp. strains	N.a.	Polyamide (PA) Polyamide/Elastane (PA/EA)	N.a.	AM: *S. epidermidis* and *C. albicans* CYT: HaCaT cells	[104]
Prodigiosin (from *Serratia marcescens*)	Red	Cotton (CO)	W 5; R 4; L 2	AM: 96.9% (*S. aureus*) UPF: Good	[133]
*Serratia marcescens*	Red	Cotton (CO) Silk (SK)	CO: W 4-5; R 4-5; L 3 SK: W 4-5; R 4-5; L 4	AM: *B. subtilis*, *E. coli*, *K. pneumoniae*, *Proteus vulgaris*, *P. aeruginosa*	[127]
*Rhodonellum psychrophilum*	Red	Cotton (CO) Viscose (CV) Silk (SK)	N.a.	AM: *E. coli*, *S. aureus*, *C. albicans*, *S. cerevisiae* Aox: 83.6%	[134]

Colour fastness (W-washing, R-rubbing, P-perspiration): (1) poor; (2) fair; (3) good; (4) very good; (5) excellent. Light fastness (L): (1) very poor; (2) poor; (3) fair; (4) moderate; (5) good; (6) very good; (7) excellent; (8) outstanding. UPF (<15) poor; (15–24) good; (25–39) very good; (40–50, 50+) excellent [126]. N.a.—not available; PAC—acrylic; PES—polyester; PA—polyamide; EA—elastane; CO—cotton; SK—silk; CV—viscose; AM—antimicrobial activity; CYT—cytotoxicity; UPF—UV protection factor; Aox—antioxidant activity.

## Data Availability

No new data were created or analysed in this study.

## Abbreviations

The following abbreviations are used in this manuscript:

AM	Antimicrobial efficacy
Aox	Antioxidant activity
BOD	Biochemical oxygen demand
CJ	Jute
CMC	Carboxymethyl cellulose
CMG	Carboxymethyl guar gum
CNC	Cellulose nanocrystals
CO	Cotton
COD	Chemical oxygen demand
CYT	Cytotoxicity
GMO	Genetically modified organisms
owf	On weight of fabric
K/S	Colour strength
MR	Mosquito repellency
PA	Polyamide
PES	Polyester
rPES	Recycled polyester
RT	Room temperature
SK	Silk
TDS	Total dissolved solids
UPF	UV protection factor
UV	Ultraviolet
WO	Wool
